# The impact of COVID-19 on online medical education: a knowledge graph analysis based on co-term analysis

**DOI:** 10.1186/s12909-023-04193-5

**Published:** 2023-04-01

**Authors:** Huijiao Deng, Yi Jiang, Qinrong Han, Xingyu Zhou, Siyang Zhong, Kai Hu, Lin Yang

**Affiliations:** 1 Zhuhai Campus of Zunyi Medical University, Zhuhai, 519000 China; 2 Key Laboratory of Human-Machine Intelligence-Synergy Systems, Shenzhen Institutes of Advanced Technology, Chinese Academy of Sciences (CAS), Shenzhen, 518055 China

**Keywords:** Online education, COVID-19, Medical education, Co-word analyse, Knowledge spectrum, Visual analysis

## Abstract

**Background:**

This study aims to identify the characteristics and future directions of online medical education in the context of the novel coronavirus outbreak new through visual analytics using CiteSpace and VOSviewer bibliometric methods.

**Method:**

From Web of Science, we searched for articles published between 2020 and 2022 using the terms online education, medical education and COVID-19, ended up with 2555 eligible papers, and the articles published between 2010 and 2019 using the terms online education, medical education and COVID-19, and we ended up with 4313 eligible papers.

**Results:**

Before the COVID-19 outbreak, Medical students and care were the most frequent keywords and the most cited author was BRENT THOMA with 18 times. The United States is the country with the greatest involvement and research impact in the field of online medical education. The most cited journal is ACAD MED with 1326 citations. After the COVID-19 outbreak, a surge in the number of research results in related fields, and ANXIETY and four secondary keywords were identified. In addition, the concentration of authors of these publications in the USA and China is a strong indication that local epidemics and communication technologies have influenced the development of online medical education research. Regarding the centrality of research institutions, the most influential co-author network is Harvard Medical School in the United States; and regarding the centrality of references, the most representative journal to which it belongs is VACCINE.

**Conclusion:**

This study found that hey information such as keywords, major institutions and authors, and countries differ in the papers before and after the COVID-19 outbreak. The novel coronavirus outbreak had a significant impact on the online education aspect. For non-medical and medical students, the pandemic has led to home isolation, making it difficult to offer face-to-face classes such as laboratory operations. Students have lost urgency and control over the specifics of face-to-face instruction, which has reduced the quality of teaching. Therefore, we should improve our education model according to the actual situation to ensure the quality of teaching while taking into account the physical and psychological health of students.

**Supplementary Information:**

The online version contains supplementary material available at 10.1186/s12909-023-04193-5.

## Background

Two thousand nineteen Coronavirus disease is a disease caused by a novel coronavirus called severe acute respiratory syndrome coronavirus [1].On December 31, 2019, WHO first learned of a cluster of pneumonia cases of the novel coronavirus reported in the city of Wuhan, People's Republic of China. This major outbreak has had a profound impact on all walks of life [2]. In particular, in the education sector, the decision to close, partially close or reopen schools should be guided by a risk management mindset, more so to maximize the education, well-being and health benefits for students, teachers, staff and the broader community, and to help prevent another outbreak of COVID-19 in the community. The decision to resume classes should be based on a careful assessment of the situation and consultation with various stakeholders, including health and education decision makers, teachers and other school staff, parents, and medical and community workers. Thus, changing the current classroom approach becomes a new and viable measure. The World Health Organization states, "If children are unable to attend school, they should be supported to ensure that students have continued access to educational materials and technologies" [3].

Online education, the Internet-based model of education in which teachers' curricula and materials are shared remotely with students through the Internet, enables the separation of teaching and learning, facilitates the flow of high-quality teaching resources, and further improves educational equity in society as a whole [4]. Since 2000, online education has become an important research direction in the field of education as an important supplement to the traditional education model. As the Internet industry continues to upgrade, the software and hardware equipment required for online teaching continues to change, making the effect of online education medical education's networking process has been ahead of other disciplines [5]. Online education in medicine has played an extremely important role for the majority of undergraduate and postgraduate teaching work and even for the continuing education of clinicians [6]. Since the novel coronavirus outbreak, social factors have contributed to the flourishing of online education, and the hotspots and directions of research have changed [7, 8].

To elucidate the impact COVID-19 has had on medical online education and to provide a systematic and objective overview of the development of medical online education, this study used Citespace, VOSviewer to separately identify, through scientometric methods, the bibliographic data published in the 10 years prior to the COVID-19 outbreak (2010–2019) and between the COVID-19 outbreak and the present day in Web of Science (WoS) journals published in the field and visualized their relationships. The analysis was based on CiteSpace. Specifically, the study was guided by four key objectives: (1) to understand the characteristics of the keywords of research topics before and after the outbreak; (2) to identify the most prominent researchers in the field before and after the outbreak and the associations between them; (3) to describe the main research institutions and the evolution of the links between geographic regions in the field before and after the outbreak; and (4) the journals with the highest number of citations and publications in the field before and after the outbreak. Each part of this paper is organized as follows. The "Materials and Methods" section describes the data collection and CiteSpace and VOSviewer, and the "Results" section provides a comprehensive analysis of the research results and their categories: research hotspots, collaborative networks, co-citation networks, etc. The "Discussion" section summarizes the history of the field of online medical education and suggests future research directions, demonstrating the broad applicability of this work. Finally, "Conclusion" section summarizes each section of the article and explains the focus of the research.

## Method

### Research overview

#### Research tools

In this study, the literature visualization and analysis tools used were CiteSpace 5.8.R3 and VOS viewer 1.6.18.

CiteSpace is a Java language-based visual literature analysis software developed by Professor Chaomei Chen of Drexel University [9]. It is able to analyze the research frontiers and research hotspots of a topic in the context of scientific metrology and data visualization [10]. Through its powerful visualization processing capability, CiteSpace has many functions such as posting volume statistics, author and institution collaboration mapping, time zone map, keyword co-occurrence map, and keyword clustering map. In CiteSpace, it counts the number of occurrences by extracting words or terms that co-occur in the same or several documents. The more co-occurrence, the closer the relationship between two subject terms or other noun terms in terms of subject content, and thus forms a co-occurrence relationship network [11].

VOS viewer is a JAVA-based software tool for building and visualizing bibliometric networks, developed in 2009 by van Eck and Waltman of The Centre for Science and Technology Studies at CWTS [12]. Its basic function and implementation principle are similar to that of citation space, but VOS viewer has better performance in specific common network mapping through co-occurrence clustering with its unique text mining techniques.

In CiteSpace, time chosen to build the dataset is selected as the time window, and the time slice is 1 year, and the text processing options of Title, Abstract, Author Keywords, and Keywords Plus are selected, and Author, Institution, and Keyword are selected as the nodes respectively The text processing options of Title, Abstract, Author Keywords, and Keywords Plus are selected, and Author, Institution, and Keyword are selected as nodes, respectively.

In the VOS viewer, you can create clusters and density diagrams by creating a bibliographic dataset based map to read the data in bibliographic database file, then filtering in the data from the scientific network and selecting co-existing, co-authorship, and other small cells in the entries to create clusters and density map that are more intuitive and clear.

#### Data sources and processing

The literature included in this paper was obtained from the Web of Science, written in English, and the Web of Science Core Collection was used as the database. In this paper, in order to better reflect the research results at different time points, the datasets were created based on the pre-COVID-19 and post-COVID-19 outbreaks, respectively. For Early Access papers published in Web of Science, we manually categorized them according to their topics, and the rest of the papers were categorized according to their publication dates.

For the literature before the COVID-19 outbreak, we chose the publication period from January 1, 2010 to December 31, 2019, 2022; the search formula was TS = online education AND medical. 4313 documents were obtained in total.

For the literature after the COVID-19 outbreak, we chose the publication dates of January 1, 2020 to July 16, 2022; the search formula was TS = online education AND medical AND COVID-19. 2555 papers were obtained in total.

The obtained documents were exported to Plain text file Full record with cited references format for analysis.

## Results

### Time distribution of the literature

**Fig.1:**
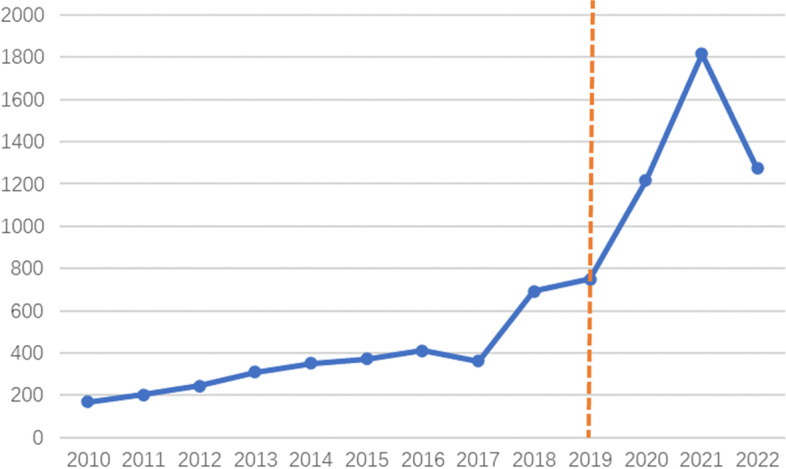
Chronological distribution of medical online education literature before and after the outbreak

The number of papers and the trend of changes can measure the level and dynamics of scientific research results in the field, which is very important for predicting future development trends. As shown in the Fig. 1, within the field of medical education, the number of online education-related papers published surged between 2019–2020, and since the literature was retrieved in October 2022 and some research papers in 2022 have not yet been published, we can reasonably speculate that the number of publications in 2022 should be significantly higher than that in 2021. Although this is influenced to some extent by information technology advances such as 5G, the role of COVID-19 in this should not be overlooked. We believe that the COVID-19 pandemic has led to a huge impact on offline teaching due to blockades of varying degrees around the world, and the huge practical demand for technological advances in online education cannot be ignored. It is foreseeable that research on online medical education will continue to increase in the future.

### Visualization of important keyword clusters analysis

An analysis of keywords in the literature for the decade prior to the epidemic (January 2010 to December 2019) and the post-epidemic period (January 2020 to June 2022) led to the following conclusions.

#### Pre-epidemic keyword cluster analysis

Scholars around the world before and after the epidemic maintained a certain level of attention to online teaching of clinical research, but after the epidemic, with the rapid development of Internet technology and the trend of the current social situation, online teaching that can communicate remotely has received extensive attention from schools [13, 14].

**Fig. 2:**
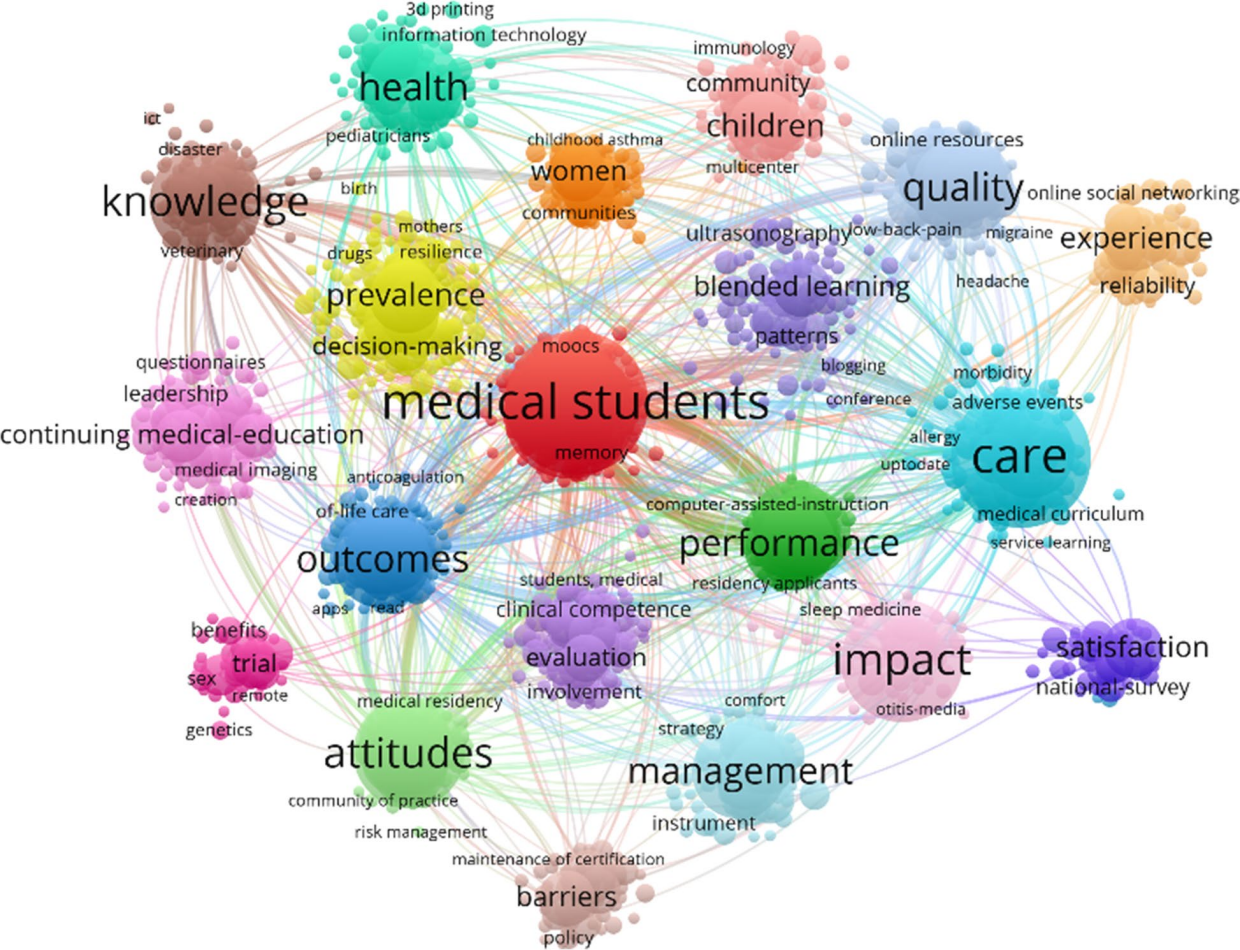
Visual clustering analysis of popular research keywords before the outbreak

A total of 10,942 keywords from the decade before the epidemic constructed a keyword network for clinical research teaching research in the decade before the epidemic. Based on the keywords in the 21 main thematic clusters, keywords with a frequency of ≥ 3 were filtered and keywords with "covid-19", "education" and their related synonyms, i.e. the terms used in the literature search for this study, were removed. We obtained 1822 keywords that were the most prominent, dominant and relevant. The visualization of the keywords drawn using VOSviewer is shown in Fig. 2.

Based on the "total link strength" indicator, it is easy to see that the following ten keywords are among the keywords with a strong link strength, which are not listed in this paper due to space, but are shown in Table 1. The term "medical student" was the most relevant term in the online teaching field during the new epidemic, with a total link strength of 2420. In addition, the keywords "care", "students" and "impact" all ranked in the top two, three and four in terms of frequency of occurrence with an association intensity of more than 1900, respectively. The phrase "curriculum" is the most frequently used term in the field of teaching and learning. The phrase "curriculum" was the fifth most frequently associated term with a strength of 1634.In terms of subject clusters, the subject clusters are ranked according to the strength of relevance and frequency of occurrence of the keywords they contain, with the first largest cluster containing 235 items, shown in red. The most frequent phrase in this cluster is "medical students", with 338 occurrences and 2420 links.

**Table 1:** 10 keywords based on total link strength before the epidemic

Keyword	Occurrences	Total link strength
Medical student	338	2420
Care	321	2257
Students	292	1984
Impact	248	1835
Curriculum	233	1634
Knowledge	213	1578
Attitude	209	1522
Quality	174	1334
Outcomes	162	1244
Health	163	1159

The second largest cluster is shown in blue in the graph and contains 157 items. The most frequent phrase in this cluster is "care", with 321 occurrences and 2257 links, making it the most frequent phrase in this cluster.

The third largest cluster contains 132 items and is shown in pink in the graph. The most frequent phrase in this cluster is "impact", with 248 occurrences and 1835 links.

The fourth largest cluster contains 117 items, highlighted in brown in the graph. Knowledge" is the most frequent phrase in this cluster, with 213 occurrences and 1578 links.

The fifth largest cluster, containing 109 items, is highlighted in light green in the graph. The most frequent phrase in this cluster is "attitudes", with 209 occurrences and 1522 links.

#### Post-epidemic keyword cluster analysis

**Fig.3:**
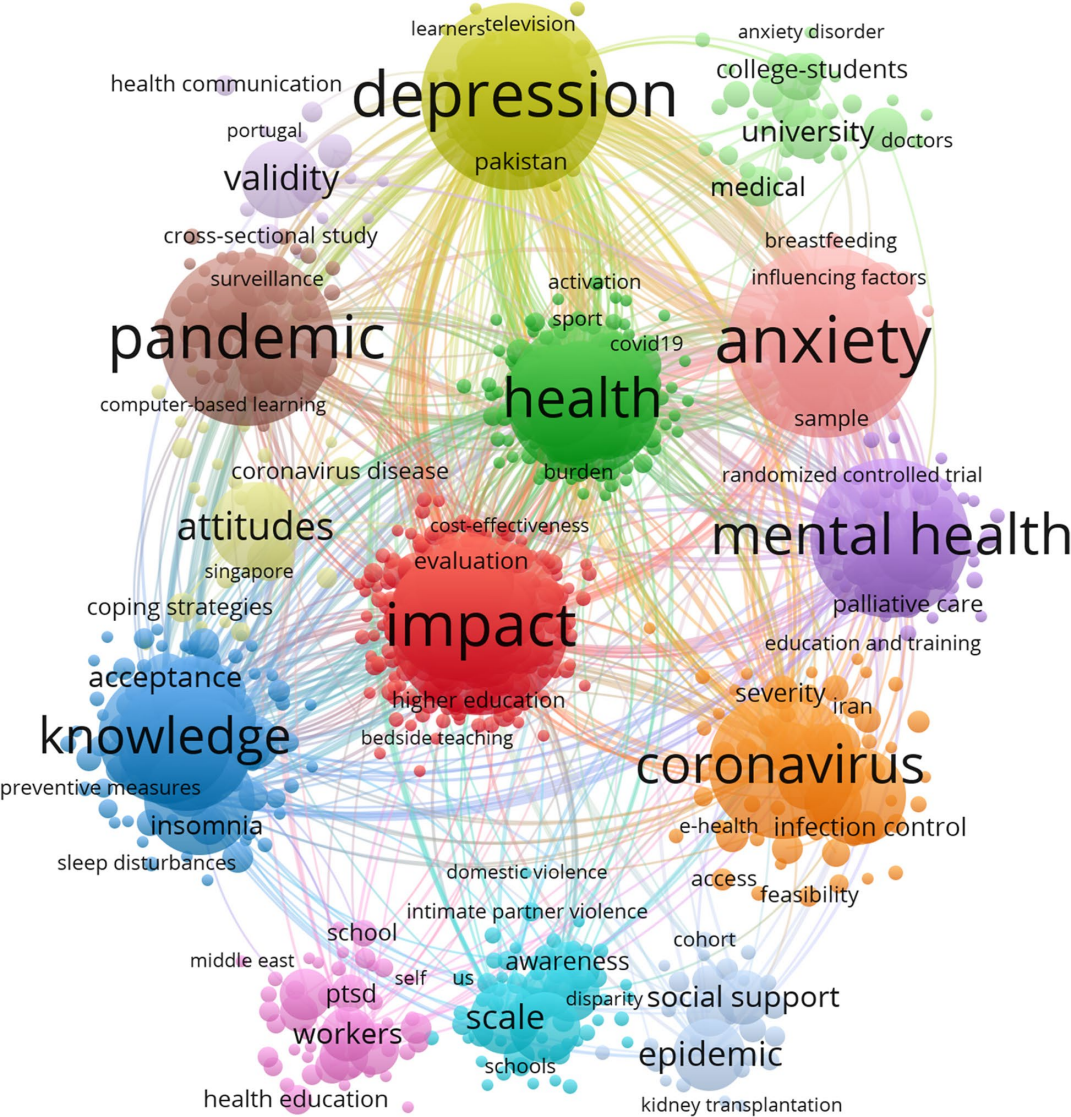
Visual clustering analysis of popular research keywords after the outbreak

A total of 6283 keywords were used to construct a keyword network for clinical research and teaching research during the novel coronavirus outbreak. Based on the keywords in the 14 main thematic clusters, keywords with a frequency of ≥ 3 were filtered and keywords with "covid-19", "education" and their related synonyms, i.e. the terms used in the literature search for this study, were removed. We obtained 1004 of the most prominent, dominant and relevant keywords. The visualization of the keywords drawn using VOSviewer is shown in Fig. 3.

Based on the "total link strength" indicator, it is easy to see that the following ten keywords are among the keywords with strong linkage, which are not listed in this paper due to space, but are shown in (Table 2). The term "Anxiety" was the most relevant term in the online teaching field during the new epidemic with a total link strength of 2138. In addition, the terms "Depression", "Impact" and "Mental health" all ranked as the most relevant terms with a total association intensity of over 1500. The keywords "Depression", "Impact" and "Mental health" are in the top two, three and four places respectively in terms of frequency of occurrence. The phrase "Stress" is in fifth place with an association strength of 1460.

**Table 2:** 10 keywords based on total link strength after the epidemic

Keyword	Occurrences	Total link strength
Anxiety	252	2138
Depression	242	2122
Impact	224	1685
Mental health	185	1506
Stress	171	1460
Pandemic	216	1441
Health	175	1210
Knowledge	140	861
Students	130	859
China	90	667

In terms of subject clusters, the subject clusters were ranked according to the strength of relevance and frequency of the keywords included. Anxiety" is the most frequent phrase in this cluster, with 252 occurrences and 2138 links.

The second largest cluster is shown in red in the graph and contains 282 items. The most frequent phrase in this cluster is "Impact", which has 224 occurrences and 1685 links.

The third largest cluster contains 57 items and is shown in brown in the graph. Pandemic" is the most frequent phrase in this cluster, with 216 occurrences and 1441 links.

The fourth largest cluster contains 141 items, highlighted in green in the graph. Health" is the most frequent phrase in this cluster, with 175 occurrences and 1210 links.

The fifth largest cluster, containing 93 items, is shown in blue. Knowledge" is the most frequent phrase in this cluster, with 140 occurrences and 861 links.

#### Comparative analysis of keyword clusters before and after the outbreak

By analyzing the top ten keywords before and after the epidemic, it is easy to find that "Students", "Impact" and "Health" are the main keywords before and after the epidemic. However, the ranking of "Knowledge" has dropped and the position of "Impact" has increased. After the epidemic, keywords such as "Anxiety" and "Stress" gradually emerged to describe psychological stress states, and the focus on students' health was gradually refined to include mental health.

### Visualization of important authors analysis

**Fig.4:**
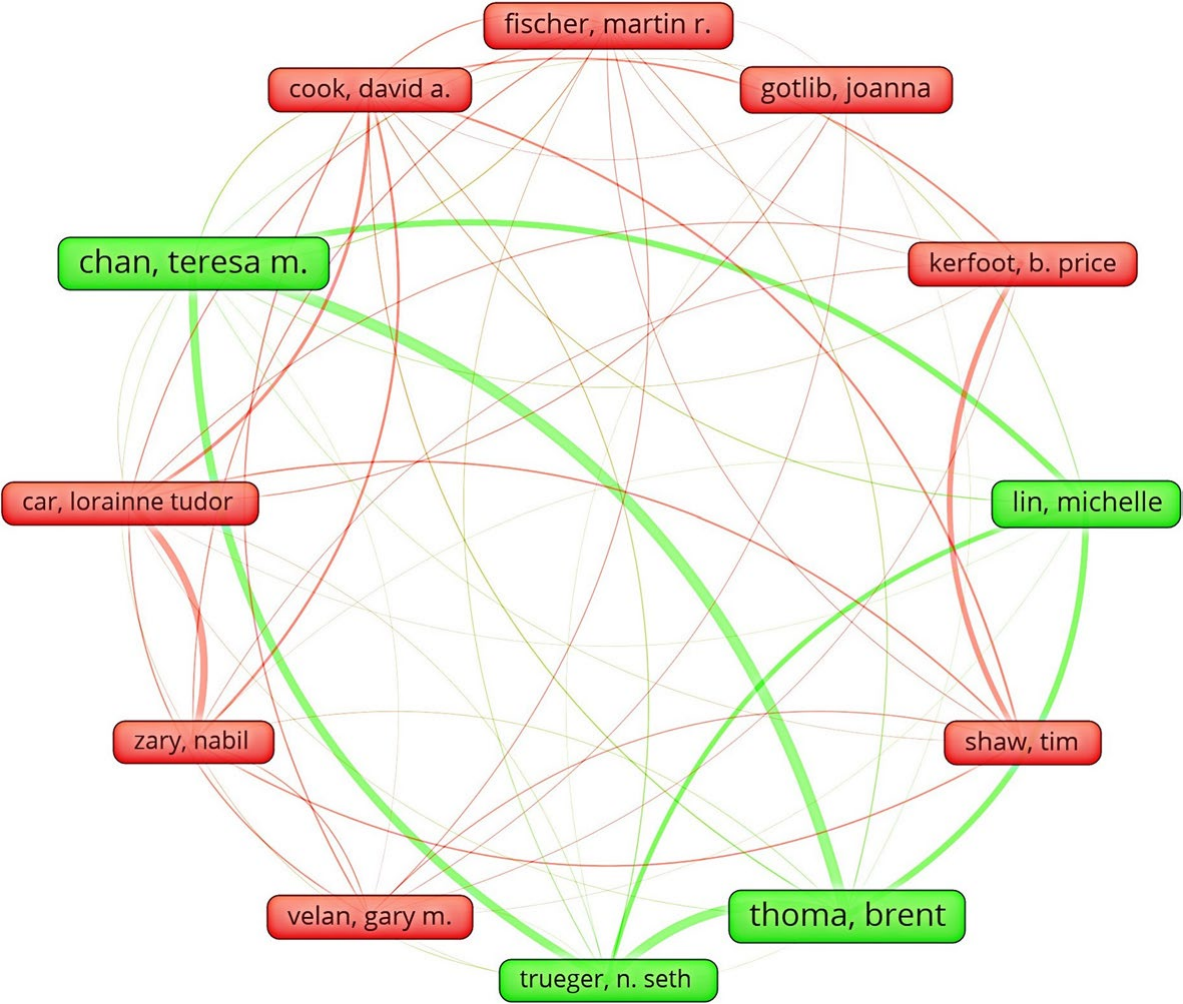
Visual clustering analysis of author coupling network before the outbreak

**Fig.5:**
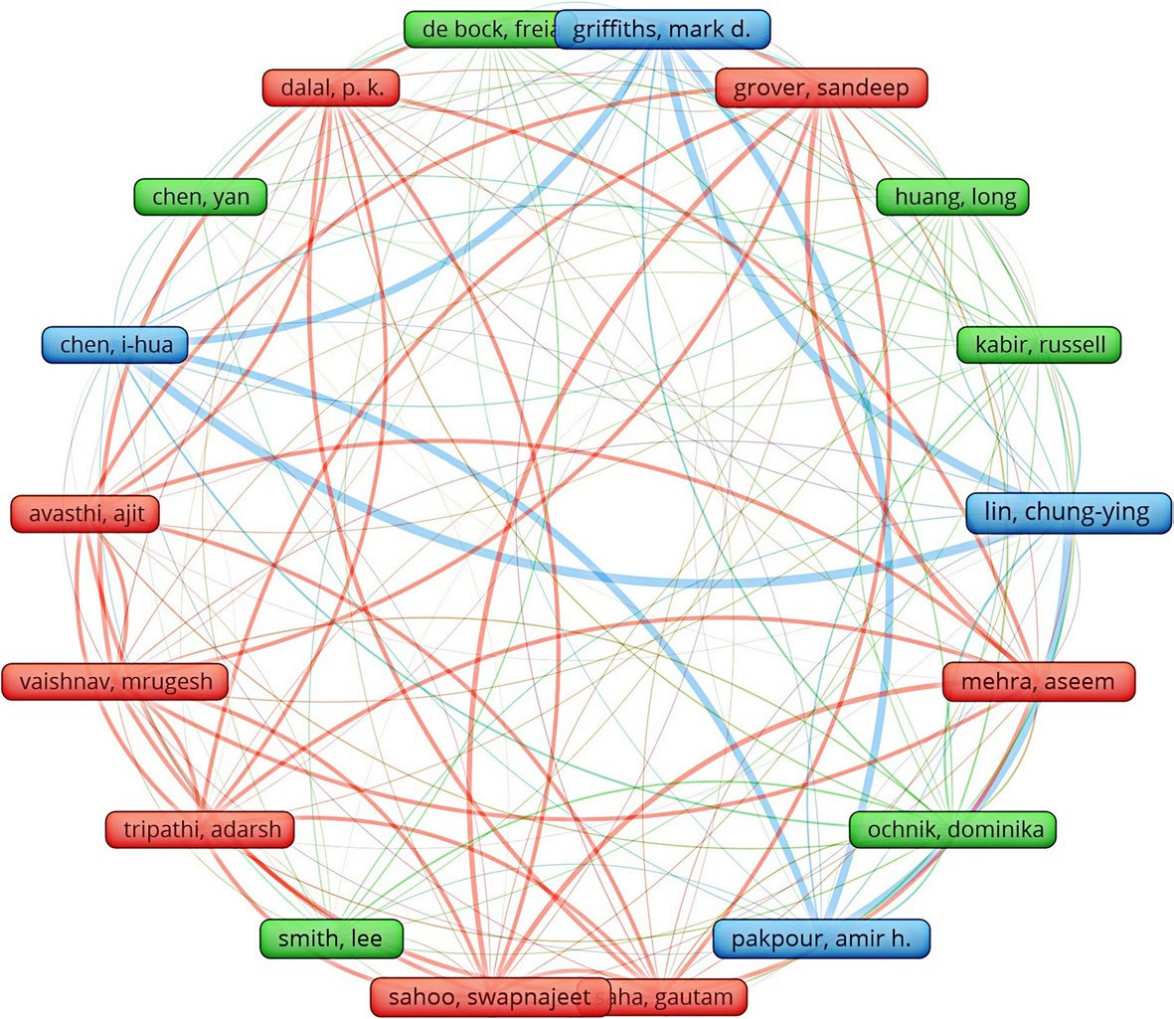
Visual clustering analysis of author coupling network after the outbreak

In the author coupling analysis before and after the epidemic using VOSviewer, the threshold was set to ≥ 7. 17 authors were obtained from 16,989 authors screened before the epidemic, and 18 authors were obtained from 15,676 authors screened after the epidemic. The deeper the curve in the figure indicates the stronger the association. From Figs. 4 and 5, we can see that although the final screening results are similar, some authors are not even shown due to clustering, indicating that some authors are not strongly associated. In general, the authors posting after the epidemic are more closely connected, and for the same topic medicine and online education, the coupling network graph of that author changes greatly after adding the topic of the epidemic, indicating the impact of the change of the research topic on the authors in the related fields.

Then using Citespace to summarize the top 10 authors of the cited articles published, as shown in the figure, before the epidemic, Brent Thoma's article was the most cited with 18 times; after the epidemic, Chungying Lin's article was the most cited with 11 times, so for a quick understanding of the field, the articles of these two are more readable (Tables 3 and 4).

**Table 3:** Top 10 citations by authors of articles published before the outbreak

Citation Counts	References
18	BRENT THOMA, 2015, SO, 0, 0
17	TERESA M CHAN, 2015, SO, 0, 0
15	NITIN AGARWAL, 2013, SO, 0, 0
13	DAVID R HANSBERRY, 2014, SO, 0, 0
8	MICHELLE LIN, 2015, SO, 0, 0
7	JOANNA GOTLIB, 2012, SO, 0, 0
7	B PRICE KERFOOT, 2010, SO, 0, 0
7	LORAINNE TUDOR CAR, 2019, SO, 0, 0
7	BERNARD T LEE, 2014, SO, 0, 0
7	CHRISTINA R VARGAS, 2014, SO, 0, 0

**Table 4:** Top 10 citations by authors of articles published after the outbreak

Citation Counts	References
11	CHUNGYING LIN, 2021, SO, 0, 0
8	FERNANDO BARBOSA, 2020, SO, 0, 0
8	LEE SMITH, 2020, SO, 0, 0
8	IHUA CHEN, 2021, SO, 0, 0
7	OMAR BOUKHRIS, 2020, SO, 0, 0
7	CHRISTIAN WREDE, 2020, SO, 0, 0
7	KHALED TRABELSI, 2020, SO, 0, 0
7	LIWA MASMOUDI, 2020, SO, 0, 0
7	ACHRAF AMMAR, 2020, SO, 0, 0
7	ASEEM MEHRA, 2020, SO, 0, 0

In terms of author centrality, pre-epidemic A Bullock, E Barnes, A Kavadella and A Liepa ranked first with a centrality of 17, while post-epidemic Aimen Khacharem and Achim Jerg ranked first with a centrality of 54, indicating the strong influence of these authors in the field (Tables 5 and 6).

**Table 5:** Top 10 posting author centrality before the outbreak

Centrality	References
17	A BULLOCK, 2013, SO, 0, 0
17	E BARNES, 2013, SO, 0, 0
17	A KAVADELLA, 2013, SO, 0, 0
17	A LIEPA, 2013, SO, 0, 0
15	E POVEL, 2013, SO, 0, 0
14	I AKOTA, 2013, SO, 0, 0
14	H KERSTEN, 2013, SO, 0, 0
14	R THOMAS, 2013, SO, 0, 0
14	S BAILEY, 2013, SO, 0, 0
14	J COWPE, 2013, SO, 0, 0

**Table 6:** Top 10 author centrality after the outbreak

Centrality	References
54	AIMEN KHACHAREM, 2020, SO, 0, 0
54	ACHIM JERG, 2020, SO, 0, 0
50	ACHRAF AMMAR, 2020, SO, 0, 0
50	ANDREA GAGGIOLI, 2020, SO, 0, 0
36	ANITA HOEKELMAMN, 2020, SO, 0, 0
21	KHADIJEH IRANDOUST, 2020, SO, 0, 0
21	ELLEN BENTLAGE, 2020, SO, 0, 0
21	KARIM CHAMARI, 2020, SO, 0, 0
21	HADJ BATATIA, 2020, SO, 0, 0
21	FAIEZ GARGOURI, 2020, SO, 0, 0

### Visualising the country density map

In this country density map showing each country's involvement in online teaching and learning during the novel coronavirus outbreak. Each point in the item density visualisation has a colour that indicates the density of the item at that point. The colours range from blue to green to yellow [15]. The greater the number of items near a point visualized in this density view, the higher the weight of neighboring items, and the closer the color of the point is to yellow. Conversely, the lower the number of items near a point, the lower the weight of neighboring items, and the closer the color of the point is to blue. From this, the most important countries in terms of online teaching and learning research engagement during the novel coronavirus outbreak period can be observed concisely and directly by plotting the visualized country densities.

**Fig. 6:**
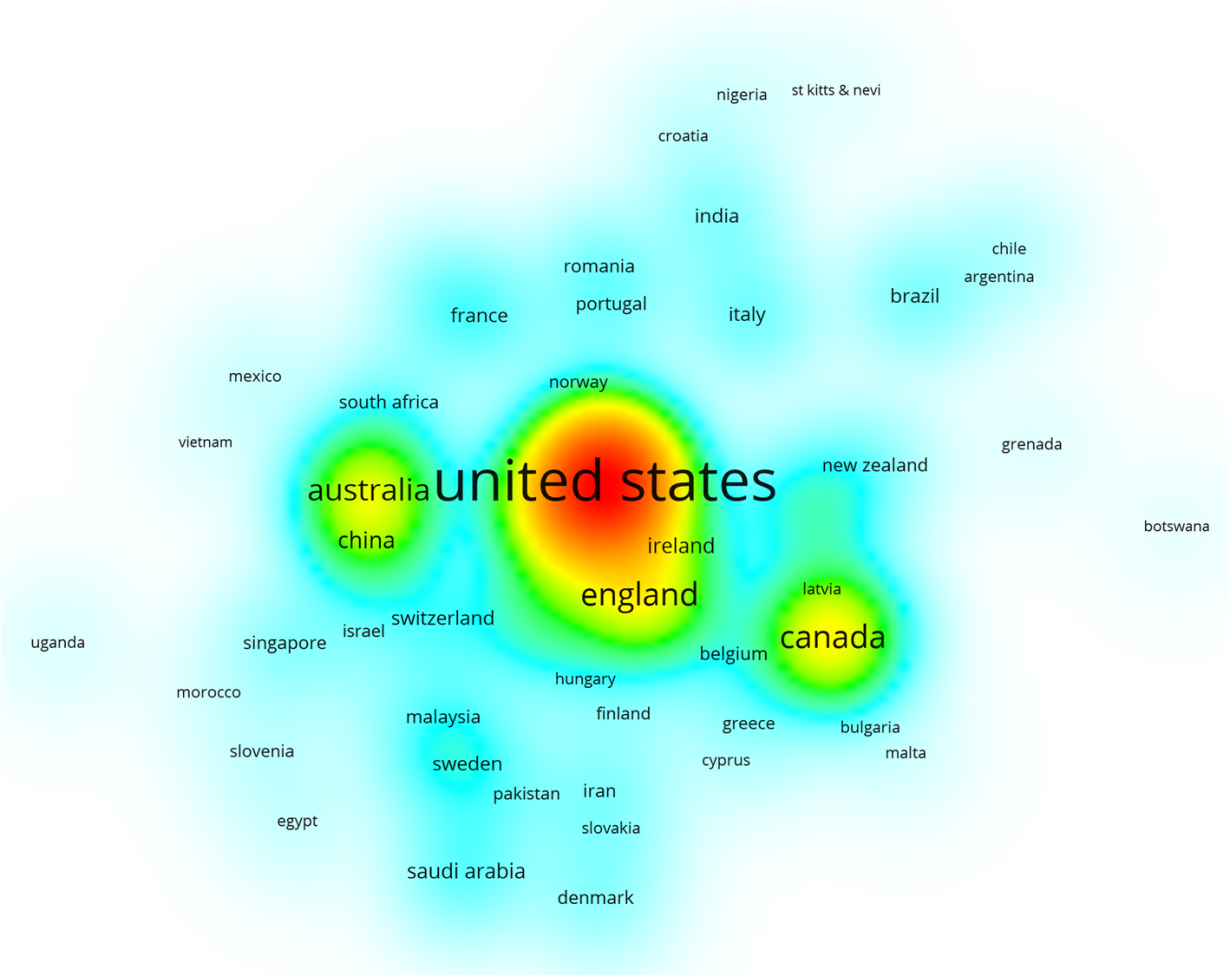
Visual clustering analysis of country density maps before the outbreak

**Fig. 7:**
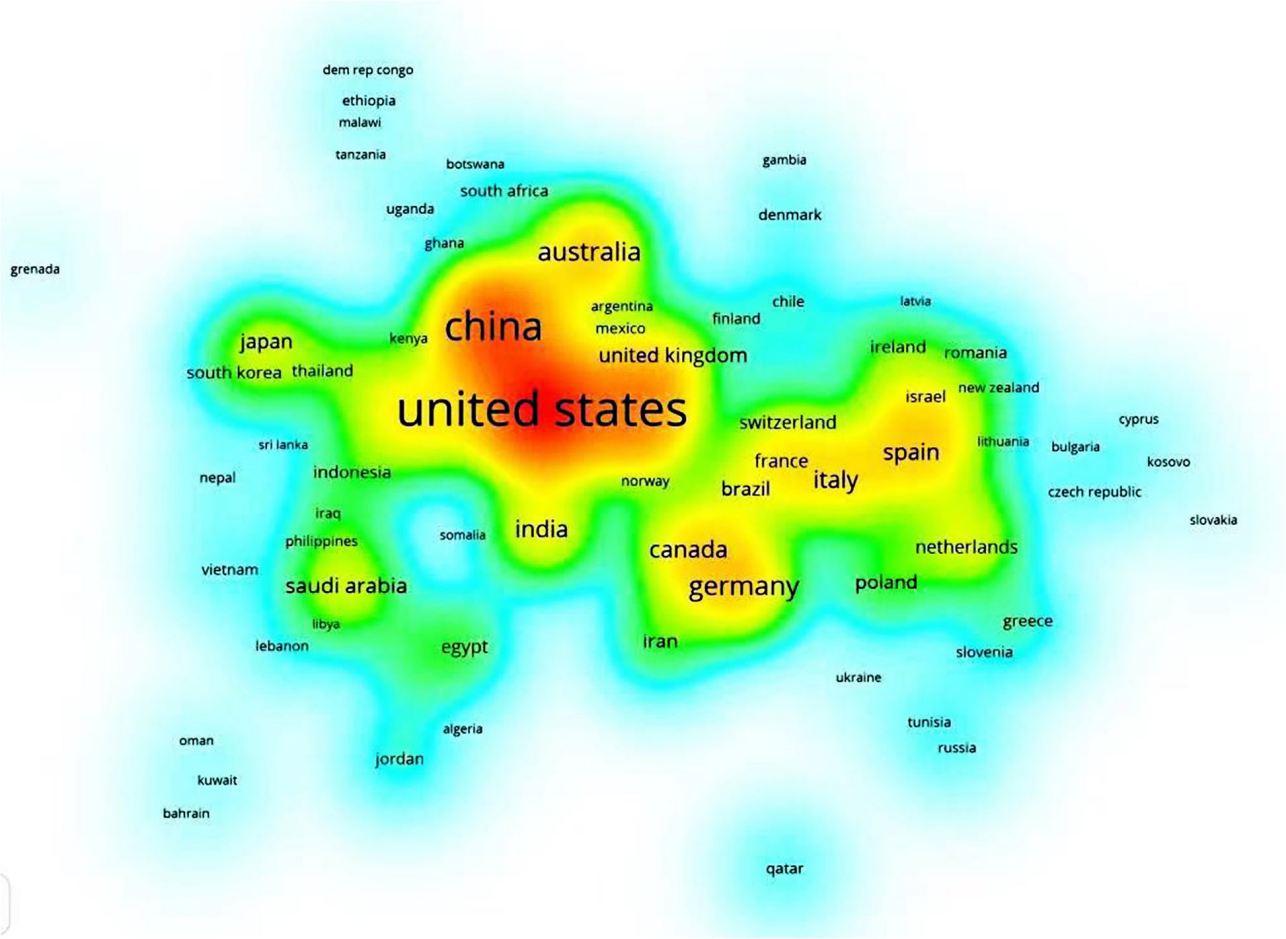
Visual clustering analysis of country density maps after the outbreak

Prior to the COVID-19 outbreak, most countries had not conducted extensive research in this area (Fig. 6). Among the major countries conducting research, the United States is the country with the greatest involvement and research impact in the field of medical online education, and researchers from the United States have closer ties with Ireland and England, China and Australia, and Canada and Latvia, but most countries are still conducting research independently without forming. The research is still conducted independently in most countries without a cooperative system. After the outbreak of COVID-19, more countries have conducted research on online medical education, but the United States is still the most influential country and has developed close ties with China and England, and countries such as Spain, Germany, Canada, Brazil, and France have also conducted a lot of research in medical online education (Fig. 7).

### Collaborative network of organizations that visualize co-authors

**Fig. 8:**
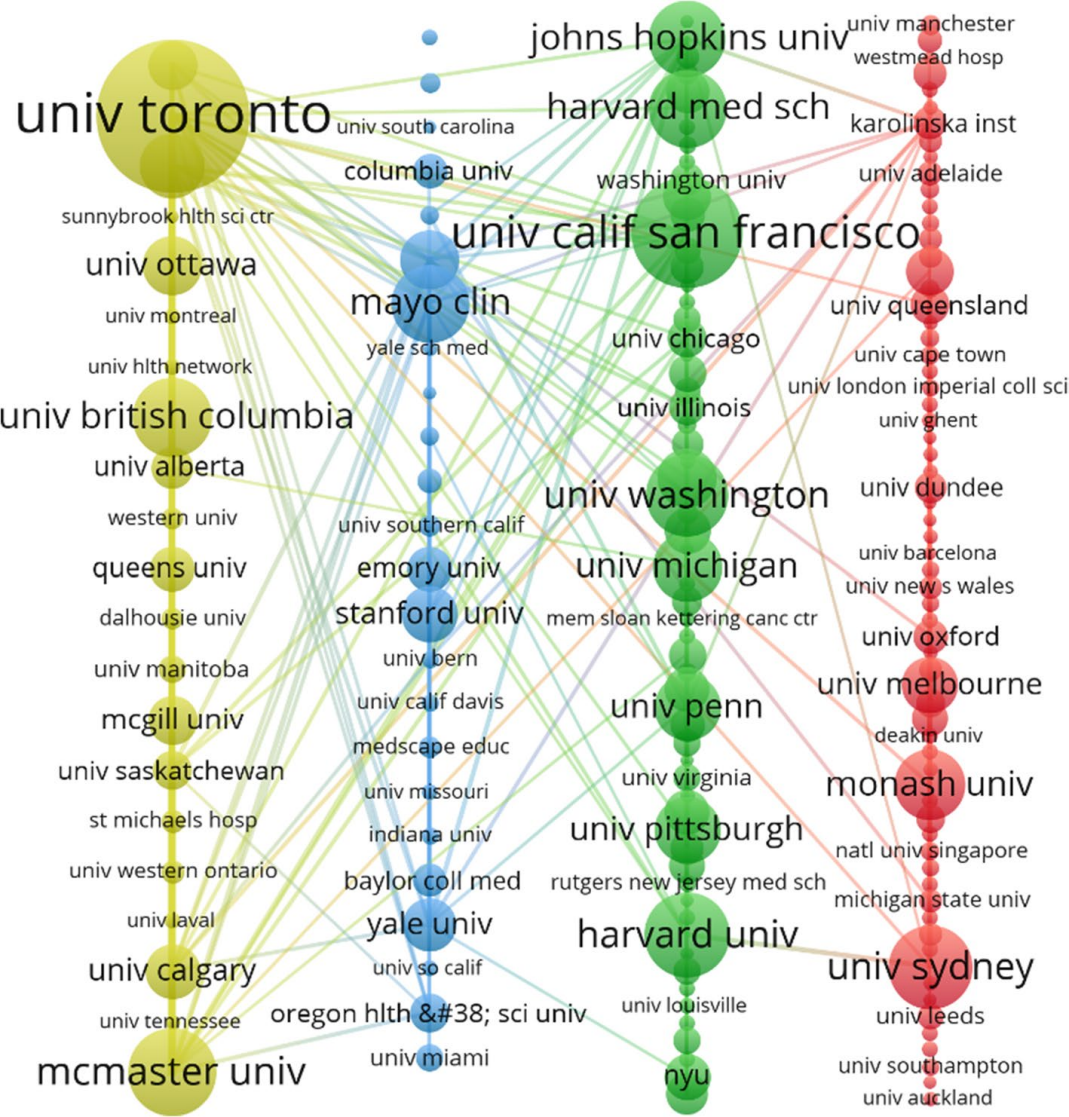
Visual clustering analysis of the institutions of co-authors before the outbreak (2) Post-epidemic organizations cluster analysis

**Fig. 9:**
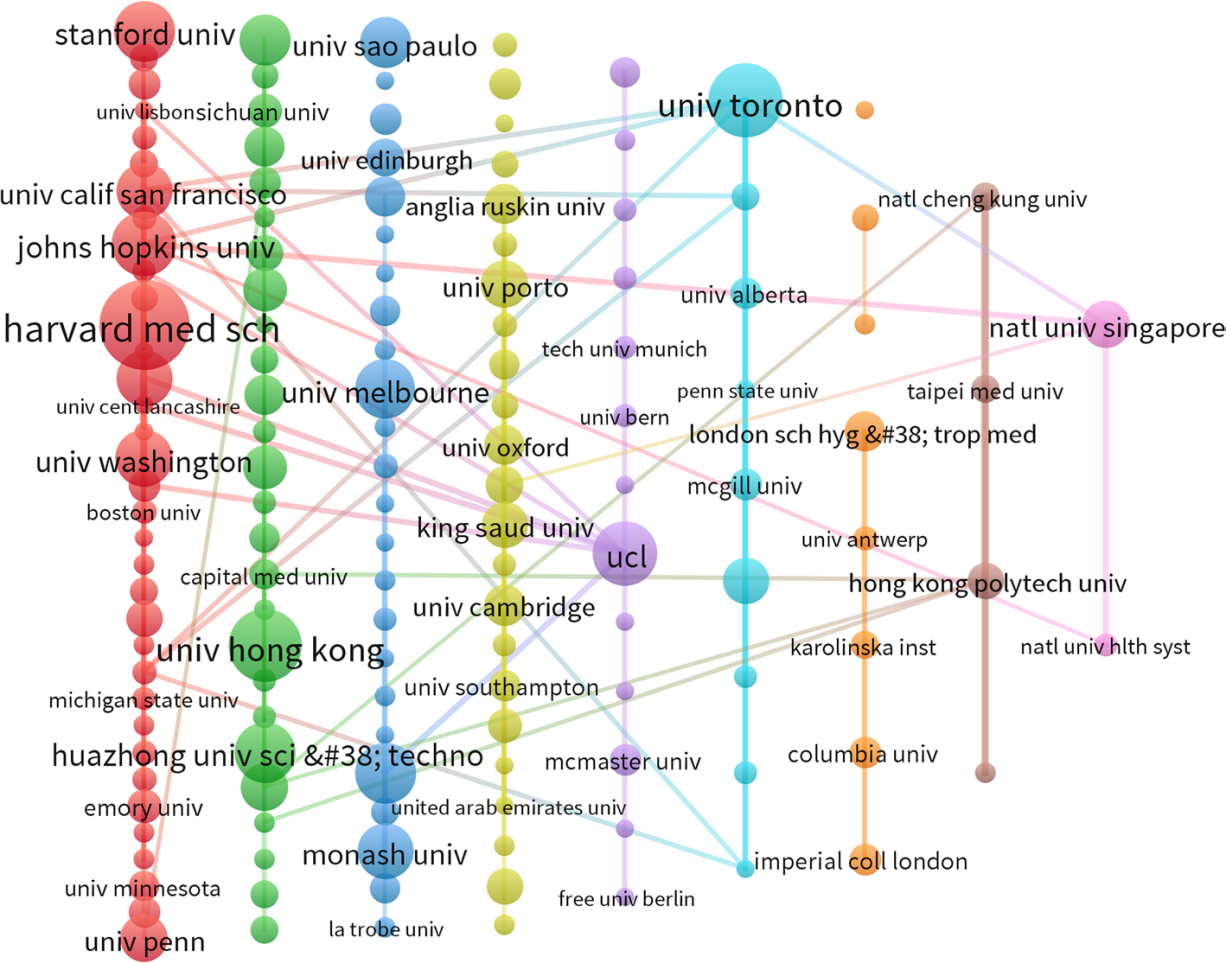
Visual clustering analysis of the institutions of co-authors after the outbreak

The visualization of the institutional collaboration network (nodes are the names of institutions that connect institutions that have collaborative relationships) provides a clear visual representation of the collaborative relationships and key institutions. (Figs. 8 and 9). The size of the nodes provides a visual representation of the centrality of each institution and the density of connections is also directly related to the closeness of the cooperation relationship.

#### Pre-epidemic organizations cluster analysis

Based on the centrality index, it is easy to conclude that "University of Toronto" is in the first place with a centrality of 2450. "Harvard University" is the second most centralised institution in the online inter-institutional collaboration network. The No. 3 institution for centrality is “University of California, San Francisco”.

According to the "Ducuments" metric, the highest-ranking institution in the visual agency collaboration network is "University of Toronto" with 115. "University of California, San Francisco" ranked second with 84, followed by "McMaster University" with 38. It is not difficult to find that the average number of "ducuments" of the top ten comprehensive literatures is about 67.

"Total link strength" refers to the Total co-occurrence times of keywords and other keywords (including repeated co-occurrence times). In the organization cooperation network of the visual co-author, it can indicate the close cooperation degree between the organization and different institutions. By analyzing the data, it's easy to know that "University of Toronto" ranks first with 20,123, "McMaster University" came in second with 17,301, and "Johns Hopkins University" came in third with 11,631. Not surprisingly, the top three are all over 10,000.

Based on the centrality index, it is easy to conclude that "National University of Singapore" is in the first place with a centrality of 3324. "Harvard Medical School" is the second most centralised institution in the online inter-institutional collaboration network. The No. 3 institution for centrality is “Huazhong University of Science and Technology”.

According to the "Ducuments" metric, the highest-ranking institution in the visual agency collaboration network is "Harvard Medical School" with 47. "

University of Toronto "ranked second with 39, followed by "The University of Hong Kong" with 38. It is not difficult to find that the average number of "ducuments" of the top ten comprehensive literatures is about 34.

"Total link strength" refers to the Total co-occurrence times of keywords and other keywords (including repeated co-occurrence times). In the organization cooperation network of the visual co-author, it can indicate the close cooperation degree between the organization and different institutions. By analyzing the data, it's easy to know that "Harvard Medical School" ranks first with 10,080. "Johns Hopkins University" came in second with 8431, and "University of Toronto" came in third with 8062.

However, considering the three indicators of "Ducuments", "Citations" and "Total link strength", it is easy to find that the development of "Harvard Medical School" in terms of publications, centrality, and total link strength with other institutions is more balanced and better. In addition, it is easy to find that the institution "Harvard Medical School" has direct links with many other institutions, which further reflects that this is a very influential institution and it maintains close cooperation with many other institutions. We realize that the outbreak of COVID-19 has produced a dramatic change in the research community in terms of online medical education. Taking this change into account in a timely manner when selecting a partner institution can go a long way in helping researchers find the right partner institution.

### Visualization of collaborative journal clustering networks

**Fig. 10:**
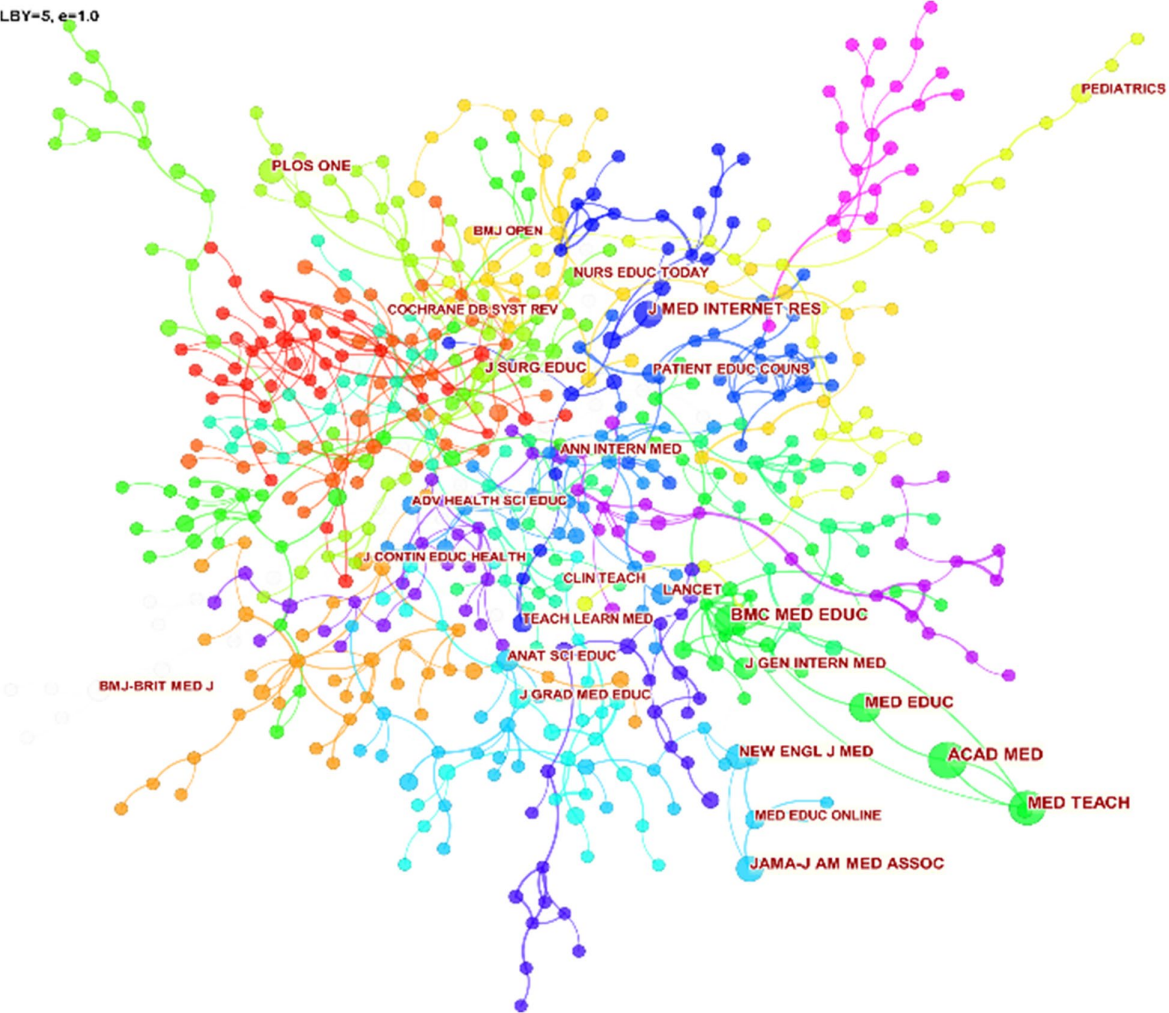
Visualizing the clustering network of journals before the epidemic

The clustering network drawn by the journals to which the visual references in this study belong (the nodes are the names of the journals, and the associated journals are connected by curves), and the clustering analysis is performed according to the main research keywords of the journals. By looking at the graphs and analyzing the data, you can explore the interconnections between journals. Specifically, the following conclusions are obtained:

The journals included in the study before the epidemic were divided into 21 clusters through cluster analysis. (Fig. 10) The journal clustering network contains a total of 770 nodes and 2647 lines. There are about 20 journals with citations above 238. By sorting the number of journal citations, the following graph (Table 7) can be obtained. This study found that the journal ACAD MED ranked first with 1326 citations, JAMA-J AM MED ASSOC was a little behind in second place with 1119 citations, and MED TEACH was third with 1080 applications. In addition, by observing the data, it can be seen that the top three citations have more than 1000 citations. In addition, MED EDUC and BMC MED EDUC are ranked fourth and fifth, respectively, with very excellent journal citations.

**Table 7:** Top 10 journals in citation counts in the clinical learning before the epidemic

Citation Counts	References
1326	ACAD MED, 2010, ACED MED, 0, 0
1119	JAMA-J AM MED ASSOC, 2010, JAMA-J AM MED ASSOC, 0, 0
1080	MED TEACH, 2010, MED TEACH, 0, 0
995	MED EDUC, 2010, MED EDUC, 0, 0
697	BMC MED EDUC, 2010, BMC MED EDUC, 0, 0
658	NEW ENGL J MED, 2010, NEW ENGL J MED, 0, 0
655	J GEN INTERN MED, 2010, J GEN INTERN MED, 0, 0
512	BMJ-BRIT MED J, 2010, BMJ-BRIT MED J, 0, 0
512	J MED INTERNET RES, 2010, J MED INTERNET RES, 0, 0
501	LANCET, 2010, LANCET, 0, 0

**Fig. 11:**
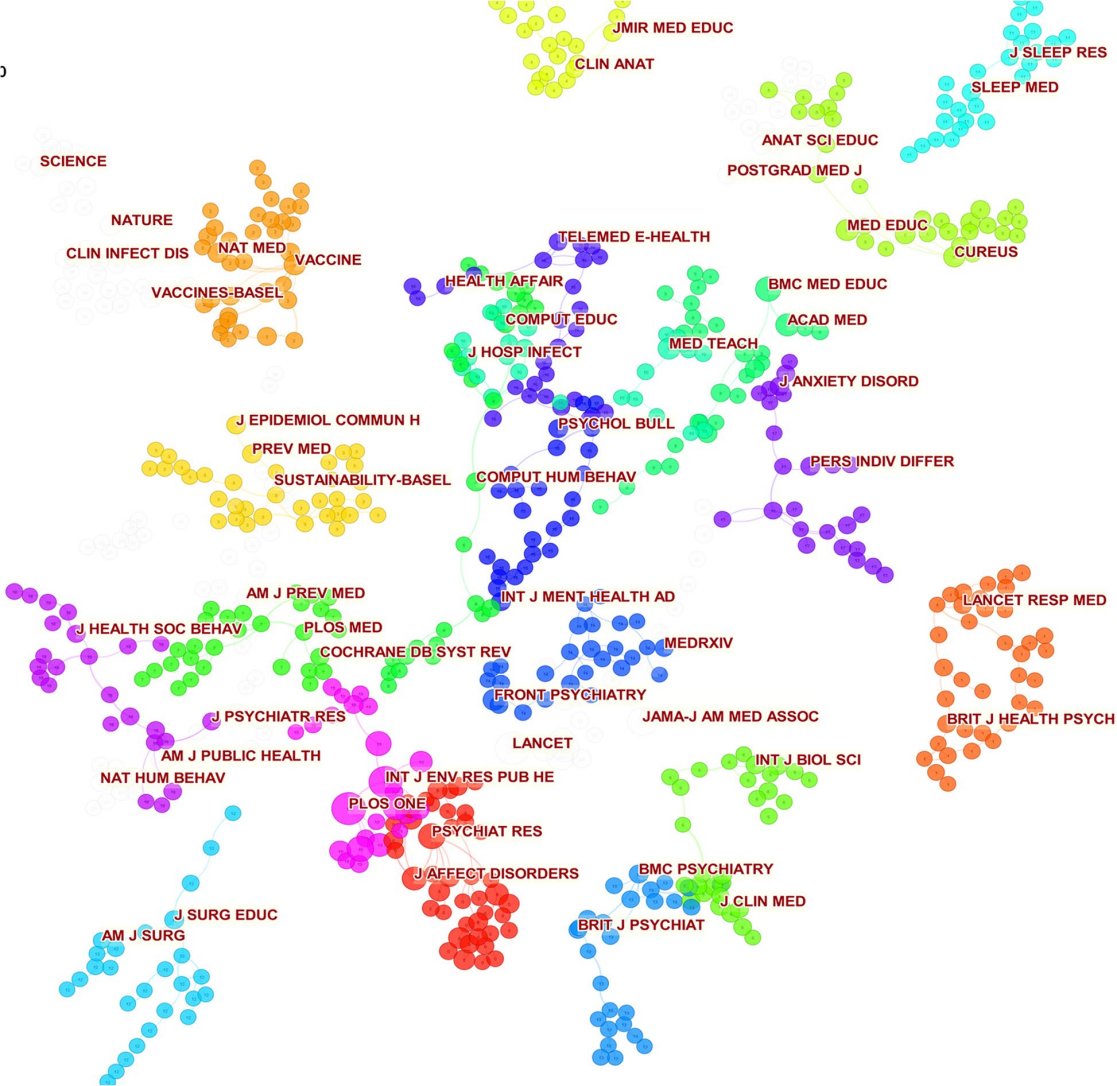
Visualizing the clustering network of journals after the epidemic

The journals included in the study after the epidemic were divided into 19 clusters through cluster analysis. (Fig. 11) By ranking the number of journal citations, the following graph can be obtained (Table 8). The journal THE LANCET was found to be in the first place with 784 citations, Public Library of Science (PLOS ONE) was slightly behind the first place in the second place with 782 citations, International Journal of Environmental Research and Public Health (INT J ENV RES PUB HE) was in the third place with 747 applications. In addition, by looking at the data it can be seen that the top three citation numbers are above 700. However, a large drop in the number of citations occurs from the fourth position.

**Table 8:** Top 10 journals in citation counts in the clinical learning after the epidemic

Citation Counts	References
784	LANCET, 2020, LANCET, 0, 0
782	PLOS ONE, 2020, PLOS ONE, 0, 0
747	INT J ENV RES PUB HE, 2020, INT J RES PUB HE, 0, 0
559	JAMA-J AM MED ASSOC, 2020, JAMA-J AM MED ASSOC, 0, 0
457	NEW ENGL J MED, 2020, NEW ENGL J MED, 0, 0
423	PSYCHIAT RES,2020, PSYCHIAT RES, 0, 0
419	J MED INTERNET RES, 2020, J MED INTERNET RES, 0, 0
394	BMJ-BRIT MED J,2020, BMJ-BRIT MED J, 0, 0
389	BMC PUBLIC HEALTH, 2020, BMC PUBLIC HEALTH, 0, 0
351	BMC MED EDUC, 2020, BMC MED EDUC, 0, 0

A comprehensive comparison between before and after the epidemic showed that 60% of the journals remained in the top 10 citation numbers after the epidemic. The analysis included "JAMA-J AM MED ASSOC" "BMC MED EDUC" "NEW ENGL J MED" "BMJ-BRIT MED J" "J MED INTERNET RES" "LANCET".

## Discussion

The essence of online education is to use Internet technology to transfer knowledge to learners through a network platform in order to achieve the purpose of cross-distance education and teaching. In addition to not being restricted by time and space, online education mode also saves time and money costs for learning to a certain extent. What's more, as a new way of education that has emerged in recent years, online education can effectively exercise students' independent learning ability and flexibility, so that learners can arrange their learning contents according to their own learning progress and interests. Medical students, because of the special nature of their profession, need to learn knowledge and accumulate experience over a long period of time, and medical education is very focused on clinical practice and hands-on. In order to meet this demand, online education combines modern intelligent visual technology to build online virtual simulation experiment simulation courses that can meet the needs of medical education. Obviously, the teaching mode of teachers and students is also facilitated by online education. In recent years, medical education and online education have shown a good trend of complementing and promoting each other. However, at the same time, online education also faces some challenges.

### Students' mental health issues

With the development of Internet and computer technologies, the technical difficulties of online teaching methods have been largely overcome, but more issues regarding students' mental health have been revealed [16]. The outbreak of Newcastle Pneumonia in early 2020 has severely impacted the normal school day. As a result of the epidemic preparedness requirements, schools have been teaching online, and it cannot be ruled out that a significant number of students will need to be educated during the quarantine period due to the Newcastle pneumonia infection. As a result, the education sector will see a massive integration of online and offline teaching and learning as the epidemic continues to progress [17]. In a study by Aidos K. Bolatov, Telman Z. Seisembekov [18], the anxiety level of medical students was somewhat increased when online education was first introduced, but was lower when fully adapted to online education. A study by Basema Saddik,1,2 Amal Hussein,1 et al. [19] on anxiety levels of clinical medicine/dental medicine students during hospital visits, before and after the introduction of online learning, noted that clinical medicine students reported higher levels of anxiety during clinical rotations, which decreased with the introduction of online learning, but non-medical students' anxiety levels increased with online learning. Similarly, a study by Jessica García-González, Wei Ruqiong, and Raquel Alarcon-Rodriguez [20] noted that the main reasons for anxiety among medical students who received online instruction were online education during a period close to graduation and being forced to live in poorer (or medically isolated) conditions for self-study. In this context, certain demands are placed on students' learning: they need to plan their studies in accordance with the requirements of the school's educational management and teachers' classroom teaching, etc., to increase their self-awareness and to overcome their fear of difficulty. On top of synchronous online learning, asynchronous online learning and independent learning are combined to achieve the best possible learning results and to alleviate anxiety [21]. At the same time, the anxiety associated with online teaching is difficult to remedy through conventional means [22]. According to one study, even with online to offline transition activities, full implementation of regular lesson plans, adequate discussion and communication in the classroom, and well-developed student–teacher feedback mechanisms, the psychological changes in students are not promising, with about a quarter of the students in the survey facing anxiety problems during online instruction [23].

### Teaching quality during online teaching

From the visual keyword clustering, it is easy to find that students/knowledge is also a hot direction of research. When students receive online education, it is difficult to generate direct communication with teachers, and it is even more difficult to realize the learning of experiments and clinical operations [24]. Meganne N. Ferrel, John J. Ryan [25] argue that replacing face-to-face courses with online courses during the COVID-19 epidemic, while the best solution for now, may also cause significant damage to education. The main impact factors are the loss of interactive and collaborative teaching models, difficulties in starting clinical apprenticeships in hospitals, and the postponement and cancellation of academic conferences [26]. In addition, this may also be related to the inadequate guarantee of teaching operation, such as the frequent "network lag", "system crash" and a series of network signal and server capacity problems during the online teaching at the beginning of the epidemic, especially in remote mountainous and less developed areas.In third world countries, not only the quality of online teaching is not promising due to these factors, but also the popularity of online teaching is not satisfactory [27]. Ahmed Alsoufi, Ali Alsuyihili, Ahmed Msherghi, Ahmed Elhadi, [28] showed that most medical students in Libya do not participate in online teaching and that the prevalence of COVID-19 significantly hinders local medical education. A study by Michał Bączek, MD, * Michalina Zagańczyk-Bączek, MD [29], pointed out that low activity in traditional courses compared to online courses and lack of interaction with patients and inadequate computer equipment were the main drawbacks of online teaching. A study by Motte-Signoret, Antoine Labbé, Grégoire Benoist [30], indicated that medical students generally believe that online teaching is not a substitute for offline free teaching and clearly oppose the continuation of online teaching after the crisis is resolved. The traditional offline teaching model is more popular and adaptable to students than the new online teaching model. Inevitably, the constraints of time and distance affect students' motivation to learn. In the future, the flexible use of online teaching and learning may make a difference. How to get students to accept and adapt to online teaching, how to make use of the advantages of online teaching, and how to strengthen the monitoring mechanism of online teaching are all things that should be taken into account in the future of online teaching [31]. As online teaching is affected by many factors and there are many problems to be solved, such as the ineffective guarantee of online teaching quality, the simple transplantation of the traditional offline classroom teaching model under the constraints of inertia, and the insufficient correlation and synergy of the core subjects and elements of online education [32]. Therefore, in the subsequent process of online education teaching reform, how to combine the advantages of traditional offline teaching mode to further explore and practice around teaching quality improvement has become an important research theme.

### Limitations

A bibliometric analysis cannot present a complete picture of the current and future state of the research field, and this study is no exception; therefore, the results of this paper are limited to its scope—that is, this paper focuses on the development of online education programs for medical students before and after the COVID-19 outbreak from 2010 to 2022 [33, 34]. However, this decade was also the period when online education developed most rapidly, especially after the outbreak of the new crown epidemic, and therefore the time frame of the data in this paper is representative; second, our study only used data obtained from Web of Science, and therefore, may have missed data that existed only in other databases (e.g., PubMed, Scopus, MEDLINE or Google Scholar) [35]. Third, due to the differences in medical education systems in different countries, the authors neglected to describe the synonymy of relevant subject terms in the construction of the dataset, resulting in the omission of some literature, which may lead to incomplete analysis results. Therefore, there is still some room for improvement in this paper, and more in-depth research can be conducted.

## Conclusion

With the development of the epidemic, the education industry, especially face-to-face medical education, which requires practice, has taken a huge hit. Online teaching is still lacking as the first method to deal with the educational problems of students affected by the epidemic. After the COVID-19 outbreak, there has been a surge in the number of relevant studies, with changes in research hotspots and in the leading researchers, institutions, countries, and the most cited journals. Analyzing a large number of academic papers can help to filter more valuable content so that the right method can be used to mitigate the negative effects of the epidemic on people.

## Supplementary Information

**Additional file 1.**

## Data Availability

All data generated or analysed during this study are included in this published article and its supplementary information files.
